# Ohio State Dental Society

**Published:** 1891-01

**Authors:** 


### Ohio State Dental Society.

DISCUSSION OF DR. CUSTER'S PAPER.

Dr. Butler: There seems to be an indiscriminate use of terms, or at least an inconsistent employment of terms, on the part of those who have introduced the ideas of dehydration or refrigeration of sensitive dentine. One claims that by throwing a fine jet of water upon a bur while it is being rotated in a cavity you get the same result that another claims for the use of heat in a cavity. It seems to be a case of blowing hot and cold out of the same mouth. If there is any virtue in having the cavity dry, I can not see the consistency of having it wet:—or vice versa. It is a very common thing that many have observed, that when the dam is applied and a cavity dried, in cases of sensitiveness of dentine, this hypersensitive condition of the dentine becomes greatly reduced. For my part I see no philosophy in the suggestion that a stream of cold water is required to keep down the heat of friction arising from the contact of so small an instrument as a bur, with the dentine of a tooth. There is a peculiar sensation not to be compared with any thing else in the cutting of the dentine of a tooth, and it is often a pretty difficult matter to convince a patient that the pain is not going to grow worse instead of becoming less. To my conception these varying processes are merely auxiliaries to assist us mainly in controlling the patient's apprehension, and in exercising his imagination. The writer of this paper points out the simplicity of the means to be employed in refrigerating the dentine, which is in contrast with the rather elaborate and complicated apparatus employed by Dr. Rhein in his process for effecting the same purpose.

Dr. Callahan: I have never tried the chloride of methy, but have used the nitrous oxide gas spray, having much difficulty to get a strong enough current. I think the imagination of the patient has much to do with the effect. I have found that by representing to the patient the effect of chloroform, leaving him to infer that I was about to use it, I could succeed in impressing —in a measure, at least—that effect upon him, while actually using alcohol instead of chloroform. Drying the cavity thoroughly, aud using sharp instruments, would perhaps have done as well as emploping the gas, unless inhalation of the gas had been employed.

Dr. Arnold: Two years ago I mentioned my use of nitrous oxide gas, suggesting its probable effect upon the imagination of the patient. I have also used a few drops of ether or chloroform, letting the patient inhale it, but not allowing him to get to any considerable extent under the influence of the drug. Nothing else compares with it. In dehydration of sensitive dentine I have found a most complete apparatus in a little invention of Dr. Niles, of Boston. It resembles an ordinary hot air syringe, and is designed to combine the vapor of alcohol with heat, in an application to the cavity of decay. I saw some very excellent results from its use. At the time I saw Dr. Niles demonstrating the use of his instrument Dr. Curtis was experimenting upon a patient in an adjoining chair, using nitrous oxide—with equally satisfactory results. My own conclusion, however, was in favor of Dr. Niles' apparatus. All this time Dr. ——, operating at a third chair, was apparently getting as good results as either of these doctors named, using same preparation in the cavity.

Dr. Niles' instrument appeared to work automatically. In buccal cavities particularly it appeared to allay sensitiveness of the dentine to a great extent. I have ordered one of the instruments for my own use.

Dr. Butler: I want to speak a word of caution as regards using chloroform even in such small quantities as a speaker has indicated. Some fatal cases of chloroform narcosis have occurred where only an infinitesimal amount of the drug was used. In fact, in the great majority of cases accompanied by alarming symptoms, the amount of the drug used was small, the patient gasping and sinking away after perhaps less than half a dozen inhalations. I speak from personal knowledge as regards these alarming symptoms. At the same time it is probable that the patient's fear of a drug which he knows to be dangerous may play some part in exercising his imagination so that he easily and unconsciously becomes a victim of his own fancy.

Dr. Arnold: It is presumed that the average practitioner has sufficient intelligence to understand the nature of this agent. I am perfectly well aware of the dangerous nature of chloroform as an agent for general administration. My idea is not to administer it in the way I have described with, any view to getting its anæsthetic effect. I want simply to gain the patient's confidence by appearing to be giving something.

Dr. H. A. Smith: This discussion reminds us that doctors will disagree. I think some of us present will recall the presence upon this floor, at a former meeting of this society, of Dr. [——?] a physician, who recommended to dentists to administer ether to the patient until he gets the "glow." I have several patients who always insist upon having ether—one lady in particular. I have no hesitation about letting her have it; she will not have cavities excavated without. I have patients whose imagination I can not impress after the manner suggested by Dr. Arnold. I would like to send some of them to him. [Laughter.] A distinguished physician of Cincinnati said that the principal difference between the quack and the reputable practitioner is that the former puts his patients to sleep—makes the patient comfortable. He is not afraid to use anæsthetics—nitrous oxide gas, chloroform, hypodermic injections, local anæsthesia, or any thing to prevent pain. And he is largely successful, too. I think the physician is right in establishing this distinction; the quack makes the patient comfortable; we let him suffer. I have seen Dr. Rhein's apparatus, but have not seen its use as applied to the teeth. My impression is that the excessive cold produced must cause intense pain to the patient. I should prefer to rely upon the administration of ether to which I have already referred.

Dr. Bollinger: I have tried many obtundents, and I agree with Drs. Custer and Arnold that to impress the imagination is as good a thing as you can do for your patient. If you can gain the patient's confidence in your purpose of handling him carefully, you can control him.

Dr. Smith: For the information of those who may wish to try Dr. Rhein's apparatus, I will state that it can be had of Messrs. McKesson & Robbins, the outfit costing about $27.50.

Dr. Butler: I have made a very good contrivance for throwing a fine jet, by using a fountain pen, drilling a small hole in the top of the holder and attaching it to a length of rubber tubing so as to allow the refrigerant spray, or whatever agent be used, to be thrown from the very fine orifice in the nose or end.

Dr. Taft: In using any of these preparations either for lowering the temperature, or for dehydration, the rubber dam should first be applied. That will prevent any unnecessary or injurious inhalation of the agent. Every one is aware that absolute dryness of the tissues favors insensibility of the dentine. All teeth can not alike be thoroughly dried. A tooth of dense structure is more thoroughly dried than one less dense. There is in the latter a diffusion of moisture that requires more time and effort to dry out. Some teeth may be expected to resist more than others the benumbing influence of cold. That depends upon the nervous susceptibility of the patient to the influence of the agent. The tooth may become benumbed under one set of conditions and not under another. We need not be surprised to find these variations. I do not think this particular agent is better than any other. Alcohol has done much in this way. The administration of various volatile agents may be effective. Caution should be used not to push to the extreme the application of cold to teeth. One of the chief objections to rhigoline was the danger of death of the pulp through the influence of the extreme cold its use produced.

[SUBJECT PASSED].

